# Evidence for acupotomology in the management of cervical radiculopathy: A protocol for systematic review and meta-analysis

**DOI:** 10.1097/MD.0000000000022007

**Published:** 2020-09-04

**Authors:** Wenkang Dai, Rui Xie, Xiongwei Wang, Minghui Zhuang, Xiaojuan Chang, Xu Wei, Zhefeng Jin, Shangquan Wang, Minshan Feng, Jie Yu, Liguo Zhu

**Affiliations:** a Wangjing Hospital of China Academy of Chinese Medical Sciences; b Beijing University of Chinese Medicine, Beijing, China.

**Keywords:** acupotomology, cervical radiculopathy, meta-analysis, protocol

## Abstract

**Background:**

Cervical radiculopathy (CR) describes compression or stimulation secondary to the cervical nerve root, 1 or 2 types of upper limb pain, and/or with neck. In clinical practice, both acupotomology and acupuncture are very widely and popular for the management of CR. So, we conducted a systematic review and meta-analysis to explore the efficacy, safety of acupotomology in the treatment of CR.

**Methods:**

We will search the following databases from inception to the September 2019 : MEDLINE(PubMed), Web of Science(Thomson Reuters), Cochrane Library, Embase (Ovid, Elsevier), SinoMed, Clinical Trials. gov, the China National Knowledge Infrastructure, Wanfang database, and VIP database. We will apply no language restrictions. We will not use a randomized controlled trial filter in EMBASE, as the set of intervention terms will limit the results sufficiently. The randomised controlled trials of acupotomology versus acupuncture for CR; two independent researchers will use the bias risk tool provided by the Cochrane Collaboration to evaluate the quality of the literature using RevMan 5.3 software (Copenhagen, The Nordic Cochrane Centre, The Cochrane Collaboration, 2014).

**Results:**

This systematic review and meta-analysis will provide a synthesis of existing evidence-based medical evidence for acupotomology/ acupotomy/needle knife in the treatment of CR.

**Conclusion:**

The conclusions of this systematic review and meta-analysis will provide evidence to evaluate the effectiveness of acupotomology/ acupotomy/needle knife for CR and further guide clinical decision-making.

**Ethics and Dissemination:**

This study is based on literature and therefore does not require ethical approval or patient consent. The study will be published in a peer-reviewed journal.

**PROSPERO registration number:**

CRD42020172274

## 1 Introduction

Cervical radiculopathy (CR), which is often due to mechanical compression, is a common neurological disorder characterized by neck and shoulder pain, weakness, or reduced reflexes.^[1]^

Physical therapy, home exercise, nonsteroidal anti-inflammatory drugs, muscle relaxers, massage, acupuncture, acupotomy are very widely and popular conservative treatments for CR management.^[2]^ A 2016 Cochrane systematic review study concluded that moderate-quality evidence suggests that acupuncture relieves pain intensity and neck disability. The review showed that acupuncture appears to be a safe treatment because of the adverse reactions are very minor.^[3]^ A acupotomy/needle knife review study concluded that acupotomy/needle knife can restore the mechanical balance of cervical vertebra, relieve spasm, and relieve pain by loosening the diseased soft tissue.^[4]^

An 2017 article from Johns Hopkins School of Medicine—Advances in the diagnosis and management of neck pain analyzed the potential benefits of muscle relaxants, nonsteroidal anti-inflammatory drugs, exercise, massage, yoga, and spinal manipulation for CR, but no mention of acupotomy/needle knife.^[5]^

Acupotomy/needle knife originated from the Nine needles in Huangdi Neijing (ancient Chinese medical books) and was developed in China by Zhu Hanzhang in 1976.^[6]^ Acupotomy/needle knife, which resembles both needle and scalpel in shape, is Traditional Chinese Medicine metal needle widely and popular used to treat cervical radiculopathy. It has a diameter of 0.4 to 1 mm and a blade width of 0.8 mm. Each acupuncturist chooses Acupotomy/needle knife with different lengths and shapes according to their personal preferences and expertise.^[7]^ On the one hand, Acupotomy/needle knife, like a acupuncture, insert into a specific “acupuncture point” stimulating analgesic substance (opioid peptides, glutamate, 5-hydroxytryptamine, and cholecystokinin octapeptide).^[8]^ On the other hand, Acupotomy/needle knife, like a scalpel, releases the trigger points or pathological sensitive points (ganglions, joints, muscles, fascia, tendons, ligaments, and so on).^[9]^

However, Acupotomy/needle knife is not clear recommended by the guidelines for the treatment of cervical radiculopathy.

So, to provide evidence-based medicine, this systematic review and meta-analysis aim to quantify the literature on acupotomy for cervical spondylosis.

## 2 Methods

### 2.1 Study registration

This protocol was registered in International Prospective Register of Systematic Reviews (PROSPERO) (registration no.CRD42020172274, which is available on https://www.crd.york.ac.uk/prospero/display_record.php?ID=CRD42020172274). The review and meta-analysis (PRISMA) method of this study and the report follow the guidelines.^[10]^

### 2.2 Inclusion criteria

#### 2.2.1 Type of studies

Published randomized controlled trials (RCT with parallel group design, cross-over design) any language will be included. We will exclude case reports, cohort studies, animal or cell experiments, review, and other non-RCTs. We will not restrict study eligibility by language or publication status.

#### 2.2.2 Type of participants

Participants of any gender or race or age or nationality with a clinical diagnosis of CRS will be included. Patients should have been diagnosed with CRS based on past or current guidelines for the diagnosis of CRS, or as defined by the trialists.

#### 2.2.3 Type of interventions

To evaluate the efficacy of acupotomy in the treatment of cervical radiculopathy. Treatment group: all studies using acupotomy or miniscapel or needle-knife as alone treatment group will be included. We will not restrict times of treatment, frequency of treatment, and length of treatment period. Other interventions will not be included in the treatment group. Control group: The control group included acupuncture, electro-acupuncture. We will not include other types of acupuncture (fine needles, fire needling, ear auricular pressure treatment, acupoint pressure, and so forth).

#### 2.2.4 Types of outcome measurements

Total effective rate, visual analogue score (VAS) 1 to 10, symptom score will be evaluated as outcomes. VAS and symptom score in the short-term or upon long-term follow-up for clinical trials. The total effective rate will be calculated based on the number of cured patients(A_1_), the number of markedly improved patients(A_2_), the number of improved patients(A_3_), and the total patients (A). Total effective rate= A_1_+A_2_+A_3_ /A.

### 2.3 Search methods

#### 2.3.1 Data sources

The search will be conducted from inception to September 2019 in the following databases: English language biomedical databases: MEDLINE(PubMed), Web of Science(Thomson Reuters), Clinical Trials. gov, Cochrane Library, Embase (Ovid, Elsevier). Chinese language biomedical databases: SinoMed, the China National Knowledge Infrastructure, Wanfang database and VIP database.

#### 2.3.2 Search strategy

The following terms will be used in the search: “acupotomy,” “acupotome,” “needle knife,” “needle scalpel,” “miniscapel-needle,” “pinknife,” “acupotomology,” “stiletto needle,” “sword like needle,” “Xiaozhendao,” “acupuncture,” “electro-acupuncture,” “cervical radiculopathy,” “cervical spondylotic radiculopathy,” “cervical spondylopathy,” “cervical spondylosis,” “neck pain,” “neck syndrome,” “nerve root cervical spondylotic,” “mechanical neck disorders,” “cervical syndrome,” “clinical trial,” “randomized trial,” “randomised trial.” Different language databases will use different search strategies.

### 2.4 Selection of studies

**Figure 1:**
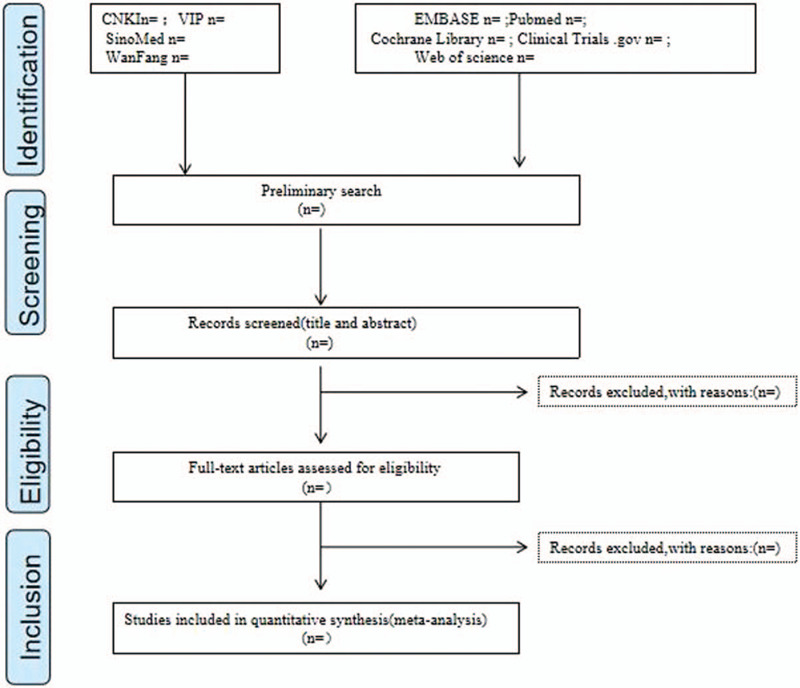
Flow diagram of study selection and screening process.

The retrieved literature will be managed by EndNoteX9 software. First of all, 2 authors will independently search the literature according to the Search strategy. Second, they will screen the titles and abstracts of all literature to exclude the literature that obviously does not meet the inclusion criteria. And then, Download and read the full text to determine whether it is included or not. If there is any disagreement, they will consult with the third independent author. The results of the search and the process of screening and selecting studies will be presented in a PRISMA flow diagram (Fig. 1)

### 2.5 Data extraction and management

Two researchers will use the standard format to extract data from the literature independently. The following data will be extracted from each trial: the publication time of the trial, the first author, characteristics of the trial population (e.g., gender, age, sample size, number of males and females), intervention measures (including the frequency and duration of interventions), duration of follow-up, outcomes assessed. If there is any disagreement, they will discuss it and consult a third review author.

### 2.6 Assessment of risk of bias in included studies

Two of our independent reviewers will use the criteria outlined in Chapter 8 of the Cochrane Handbook for Systematic Reviews of Interventions to assess the bias risk of all included studies. Information on risk of bias will be added to the “Risk of bias” tables in RevMan 5.3 software (Copenhagen, The Nordic Cochrane Centre, The Cochrane Collaboration, 2014). We will assess the risk of bias according to the following domains: random sequence generation; allocation concealment; blinding of participants and personnel; blinding of outcome evaluation; incomplete report outcome; selective outcome reporting; other biases. They will classify the risk of bias as “Low risk,” “High risk,” and “unclear risk” in each domain. They will provide bias graph and graphic summary of risk of bias in the completed review. If there is any disagreement, a third reviewer will intervene, and consensus will be reached by discussion.

### 2.7 Measures of treatment effect

Dichotomous outcomes (e.g., total effective rate) will be analyzed by calculating the relative risk. Continuous outcomes (e.g., visual analog scale) will use mean difference as the effect analysis statistics. If the same outcome is measured by different ways, the standardized mean difference will be used to measure outcomes. Assessment of heterogeneity: I^2^ statistic and *χ*^2^ test will be used to assess potential heterogeneity in the studies. When inconsistency is of low importance (*P* > .1 or I^2^≤50%), we will use a fixed effect model for meta-analysis. When inconsistency is judged to be substantial (*P* <.1 or I2 > 50%), we will analyze data using a random-effects model.

### 2.8 Sensitivity analysis

We will assess the reasonableness of pooling on clinical grounds. We will analyze the possible sources of heterogeneity: intervention type, characteristics of treatment (including the frequency and duration of interventions), symptom duration.

### 2.9 Reporting bias analysis

When >10 L are included and visual asymmetry on the funnel plots, We will use funnel chart and Egger regression analysis to assess publication bias.

## 3 Discussion

Cervical radiculopathy is most prevalent in persons 50 to 54 years of age.^[11]^ The annual age-adjusted incidence rate was 83.2 per 100,000 persons.^[12]^

Acupotomy/needle knife has both acupuncture effect and microinvasive operation effect. On the one hand, Acupotomy/needle knife activates afferent fibers (Abeta, Adelta, and C-fos),^[13]^ stimulates different signaling molecules (release of neurotransmitters, endogenous opioid-like substances) to mediate acupuncture analgesia.^[14]^ On the other hand, Acupotomy/needle knife can relieve soft tissue adhesion, muscle tension, and muscle spasm, improve local microcirculation, and restore biomechanical balance of cervical vertebra.^[15]^

In clinical practice, most patients with cervical radiculopathy will improve with nonoperative (such as Acupotomy/needle knife and so on). Due to the lack of high-quality literature, there are no clear recommendations in the guidelines for the treatment of cervical radiculopathy by acupotomy/needle knife.

So, we hope that the results of this study can contribute to acupotomy/needle knife treatment of cervical radiculopathy and provide evidence-based medical evidence.

## Author contributions

**Conceptualization:** WD, LZ.

**Data curation:** XW, RX.

**Investigation:** MZ, XC.

**Methodology:** ZJ, SW.

**Resources:** MF, XW.

**Software:** XC, RX.

**Supervision:** JY, LZ.

**Writing – original draft:** WD.

**Writing – review & editing:** WD, JY, LZ.
